# Phosphorylation of ERK in neurokinin 1 receptor-expressing neurons in laminae III and IV of the rat spinal dorsal horn following noxious stimulation

**DOI:** 10.1186/1744-8069-3-4

**Published:** 2007-02-19

**Authors:** Erika Polgár, Annie D Campbell, Lynsey M MacIntyre, Masahiko Watanabe, Andrew J Todd

**Affiliations:** 1 Spinal Cord Group, Institute of Biomedical and Life Sciences, University of Glasgow, Glasgow G12 8QQ, UK; 2 Department of Anatomy, Hokkaido University School of Medicine, Sapporo 060-8638, Japan

## Abstract

**Background:**

There is a population of large neurons with cell bodies in laminae III and IV of the spinal dorsal horn which express the neurokinin 1 receptor (NK1r) and have dendrites that enter the superficial laminae. Although it has been shown that these are all projection neurons and that they are innervated by substance P-containing (nociceptive) primary afferents, we know little about their responses to noxious stimuli. In this study we have looked for phosphorylation of extracellular signal-regulated kinases (ERKs) in these neurons in response to different types of noxious stimulus applied to one hindlimb of anaesthetised rats. The stimuli were mechanical (repeated pinching), thermal (immersion in water at 52°C) or chemical (injection of 2% formaldehyde).

**Results:**

Five minutes after each type of stimulus we observed numerous cells with phosphorylated ERK (pERK) in laminae I and IIo, together with scattered positive cells in deeper laminae. We found that virtually all of the lamina III/IV NK1r-immunoreactive neurons contained pERK after each of these stimuli and that in the great majority of cases there was internalisation of the NK1r on the dorsal dendrites of these cells. In addition, we also saw neurons in lamina III that were pERK-positive but lacked the NK1r, and these were particularly evident in animals that had had the pinch stimulus.

**Conclusion:**

Our results demonstrate that lamina III/IV NK1r-immunoreactive neurons show receptor internalisation and ERK phosphorylation after mechanical, thermal or chemical noxious stimuli.

## Background

The neuropeptide substance P is expressed by many nociceptive primary afferents that innervate skin and deeper tissues, and is contained within their central terminals in the superficial laminae of the dorsal horn [1-4]. Substance P is released from these terminals following noxious stimulation [5,6] and acts on neurokinin 1 receptors (NK1rs) that are present in the plasma membranes of certain neurons in the dorsal horn. Cells that possess high levels of the NK1r are most numerous in lamina I, but there is also a population of large NK1r-immunoreactive neurons that have their cell bodies in lamina III or IV and dendrites that pass dorsally to enter lamina I [7-12]. Approximately 20 cells of this type are present on each side in the L4 spinal segment in the rat [13].

Dorsal horn neurons with long axons that ascend in the white matter and project to the brain (projection neurons) are present in relatively large numbers in lamina I and are scattered throughout the deeper laminae (III-VI). The great majority (~80%) of projection neurons in lamina I express the NK1r [13-17], and the large NK1r-immunoreactive cells in laminae III-IV are also known to be projection neurons, since virtually all of them can be labelled following injection of tracer into the caudal ventrolateral medulla [13]. We have shown that the large lamina III/IV NK1r-expressing cells are strongly innervated by substance P-containing primary afferents, which form numerous synapses on their dendrites and cell bodies [18]. This suggests that they would be strongly activated by noxious stimulation. However, Torsney and MacDermott [19] carried out whole-cell recordings from spinal cord slices *in vitro *and were unable to demonstrate monosynaptic inputs from C or Aδ primary afferents for the majority of lamina III cells that expressed NK1rs. In addition, Doyle and Hunt [20] reported that while 40% of these cells up-regulated the transcription factor fos in response to a subcutaneous injection of formalin, few of them expressed fos after other types of noxious stimulus, including noxious thermal stimulation.

Several immunocytochemical studies have demonstrated activity-dependent phosphorylation of extracellular signal-related kinases 1 and 2 (ERK1/2) in the spinal dorsal horn after various types of noxious stimulus or nerve injury *in vivo*, and after electrical stimulation of Aδ/C primary afferent fibres *in vitro *[21-29]. It has been shown that following either acute noxious stimulation or activation of fine diameter primary afferents, phosphorylated ERK (pERK) is present in many neurons located in laminae I-II, as well as in scattered cells in deeper laminae. However, little is known about the types of neuron that contain pERK, except that 24 hr after injection of complete Freund's adjuvant into the hindpaw, most neurons in lamina I that contained prodynorphin or possessed NK1 receptors were pERK-immunoreactive [23]. The aim of this study was to determine whether the large NK1r-immunoreactive neurons in laminae III-IV of the dorsal horn contained pERK after various types of acute noxious stimulus. We find that following noxious mechanical, thermal or chemical stimulation of one hindpaw, virtually all of the cells of this type that were located in the medial part of the ipsilateral dorsal horn showed pERK-immunoreactivity.

## Results

### pERK staining

**Figure 1:**
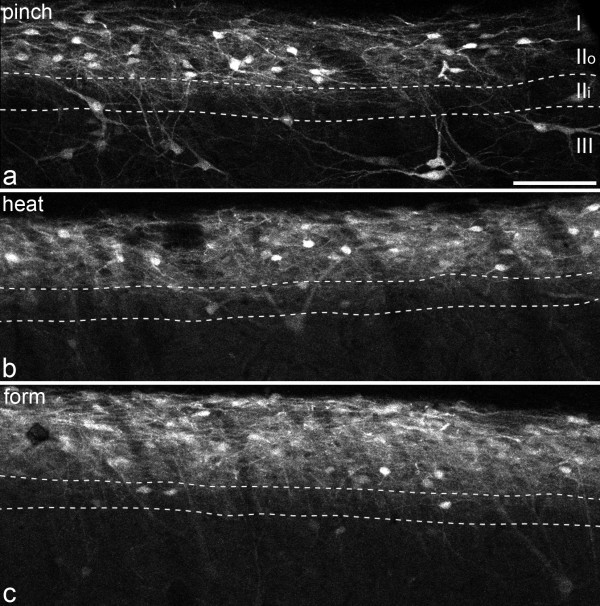
**Phosphorylation of ERK in the ipsilateral dorsal horn after different types of noxious stimulus**. pERK-immunostaining in confocal images of parasagittal sections through the medial part of the left dorsal horn from rats that were perfused with fixative 5 mins after the end of different types of noxious stimulus: **a **repeated pinching of the skin of the foot for 1 min (pinch), **b **immersion of the hindpaw in water at 52°C for 1 min (heat), **c **injection of 100 μl 2% formaldehyde into the hindpaw (form). These sections were also immunostained for PKCγ (not shown) and the two dashed lines, which outline lamina IIi, were drawn from the location of the plexus of PKCγ-immunoreactive dendrites (for further details see text). The positions of laminae I, IIo and III are also indicated in **a**. In all cases there is strong pERK-immunoreactivity that is mainly restricted to laminae I and IIo. Some pERK-immunoreactive cells were seen ventral to the IIo/IIi border, particularly after pinch. All images are projections of z-series consisting of 10 optical sections at 2 μm spacing. Scale bar = 100 μm.

One of three types of noxious stimulus was applied to the left hindpaw of anaesthetised rats: repeated pinching of the skin for 1 minute (n = 4), immersion of the paw in water at 52°C for 1 min (n = 3), or subcutaneous injection of 100 μl 2% formaldehyde (n = 3). Five minutes after the end of each type of stimulus, numerous pERK-immunoreactive cells and processes were visible in the medial part of the ipsilateral dorsal horn in the L4 spinal cord segment (Fig. 1). In each case, these were highly concentrated in a band that occupied lamina I and the outer (dorsal) half of lamina II (lamina IIo). We have previously shown that a plexus of PKCγ-immunoreactive dendrites in the superficial dorsal horn occupies the inner (ventral) half of lamina II (IIi) [30], and the dorsal and ventral borders of this plexus were therefore used on some sections in the present study to define the boundaries between lamina IIo/IIi and lamina IIi/III, respectively. Scattered pERK-positive cells were present in the deeper laminae after each type of stimulus, and although not quantified, these appeared to be more numerous in animals that had undergone the pinch stimulus than those that had received noxious thermal or chemical stimulation (Fig. 1). A few cells with weak pERK-immunoreactivity were seen in the superficial dorsal horn in the lateral half of the ipsilateral side and on the contralateral side. However, the lamina III/IV NK1r-immunoreactive neurons in these regions were never pERK-positive.

### pERK and NK1r

**Figure 2:**
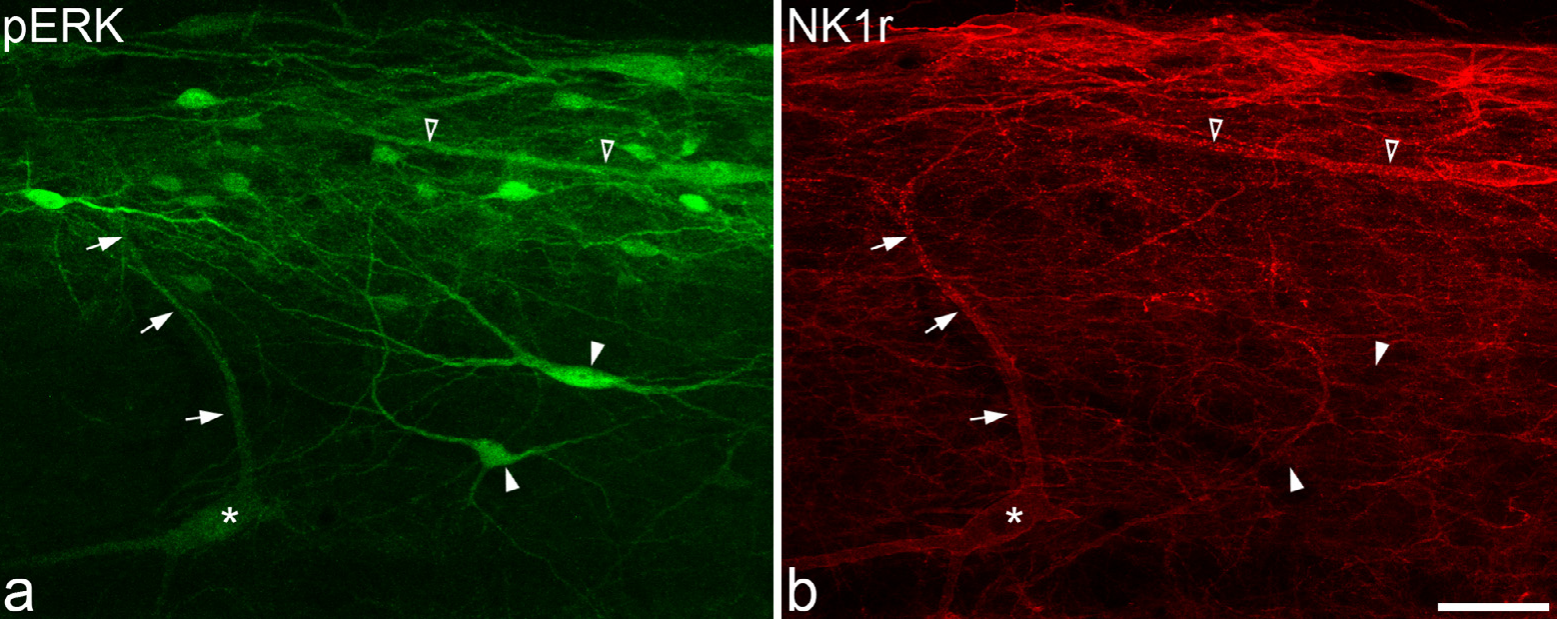
**pERK and NK1r following pinch**. These confocal images show **a **pERK- and **b **NK1r-immunoreactivity in a parasagittal section of the left dorsal horn following pinch stimulation. **a **Many pERK-positive profiles are present in a band that corresponds to laminae I and IIo. In **b**, the cell body of a large NK1r-positive lamina III neuron is indicated with an asterisk, and one of its dorsal dendrites is shown with arrows. The dorsal half of this dendrite (dorsal to the middle arrow) shows internalisation of the NK1r. The cell body and dorsal dendrite of the neuron are weakly immunoreactive for pERK (**a**). Two other pERK-positive cells in lamina III are indicated with arrowheads. These are not NK1r-immunoreactive, but have dorsal dendrites that extend at least as far as lamina II. The open arrowheads point to the dendrite of a large NK1r-immunoreactive lamina I neuron that has internalised receptor (**b**) and pERK-immunostaining (**a**). Images are projections of 7 optical sections at 2 μm z-spacing. Scale bar = 50 μm.

**Figure 3:**
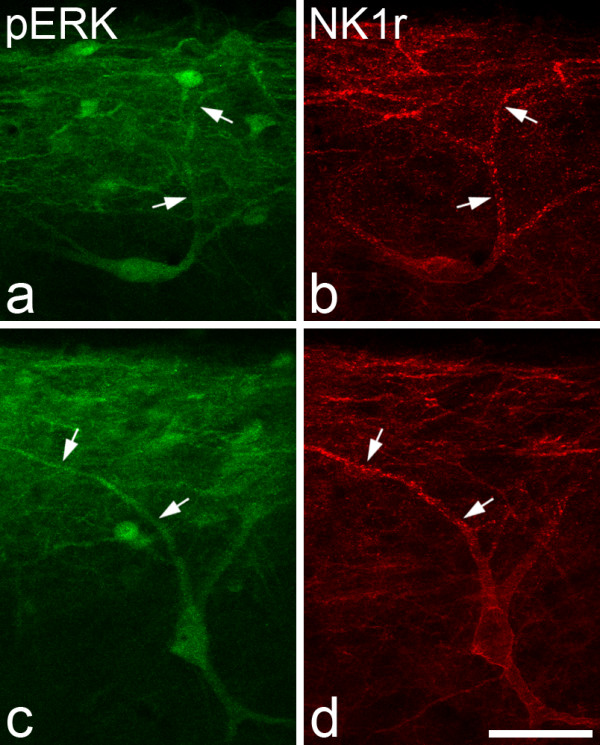
**pERK and NK1r following noxious thermal stimulation or formaldehyde injection**. Confocal images that show immunoreactivity for pERK (**a,c**) and NK1r (**b,d**) in parasagittal sections of the left dorsal horn following immersion of the hindpaw in water at 52°C (**a**,**b**) or injection of 2% formaldehyde (**c**,**d**). In each case there is a single large lamina III NK1r-immunoreactive neuron that is also pERK-positive, and there is internalisation of the receptor on its dorsal dendrites (arrows). The images are projections of 11 (**a**,**b**) or 8 (**c**,**d**) optical sections at 2 μm z-spacing. Scale bar = 50 μm.

The laminar distribution of NK1r-immunostaining was the same as that described previously [7-12]. The highest density of immunostained profiles (both cell bodies and dendrites) was present in lamina I, and scattered large NK1r-positive neurons were located in laminae III and IV. In most cases the dendrites of these deep cells could be followed into the superficial dorsal horn, either on the same section or on an adjacent section. After all types of stimulus there was extensive internalisation of NK1rs, which gave rise to numerous endosomes in the cell bodies and dendrites of lamina I neurons, and in the dorsal dendrites of the large lamina III/IV cells (Figs. 2, 3).

Many, but not all, of the NK1r-immunoreactive lamina I neurons in the medial part of the ipsilateral dorsal horn showed internalisation of the receptor and the great majority of these were pERK-positive (Fig. 2). In some cases, lamina I neurons with the NK1r that did not appear to have significant internalisation showed pERK-immunoreactivity. However, we cannot rule out the possibility that there was internalisation in some parts of the dendritic trees of these cells. Very rarely, we saw lamina I cells with clear NK1r internalisation that did not appear to be pERK-immunoreactive. Again, it is possible that there could have been phosphorylation of ERK in distal dendrites of these cells. pERK-immunostaining was also present in many cells that were not NK1r-immunoreactive in laminae I and IIo.

The number of large lamina III/IV NK1r-immunoreactive cells that were identified in the sections from the medial part of the ipsilateral dorsal horn in the 10 rats varied from 6–13 (mean 9.4). In all cases, dorsal dendrites of these cells could be followed at least as far as lamina II. Their cell bodies were located between 100 and 281 μm (mean 209 ± 44, SD) below the dorsal white matter. After each type of stimulus, pERK-immunoreactivity was detected in virtually all (97–100%) of these neurons (Table 1, Figs. 2, 3). The intensity of pERK-immunostaining in these cells varied from very weak to moderately strong. In most (72/92) of the pERK-positive lamina III/IV NK1r neurons, pERK-immunoreactivity was detected in both cell body and dendrites, while in the remaining 20 neurons only the dorsal dendrites were immunoreactive (Table 1). Even in the cells that showed pERK-immunostaining in both soma and dendrites, the staining was often considerably stronger in the distal parts of the dorsal dendrites (Fig. 2a). On the great majority of these neurons, NK1r-internalisation was detected in at least some of the dorsal dendrites that lay within lamina I and II. However, for all of the pERK-positive cells the pERK-immunoreactivity was not restricted to dendrites that showed internalisation.

**Table 1:** pERK in lamina III/IV NK1r-immunoreactive neurons

Stimulus	Number of lamina III/IV NK1r-immunoreactive cells examined	Number positive for pERK	Number with somatic pERK
pinch (n = 4)	32	31 (97%)	26 (81%)
heat (n = 3)	28	28 (100%)	25 (89%)
formaldehyde (n = 3)	34	33 (97%)	21 (62%)

This shows the total number of NK1r-immunoreactive neurons in laminae III or IV that were examined in rats that had received each type of stimulus, together with the number (and percentage) that contained pERK and the number (and percentage) that had detectable pERK-immunoreactivity in their cell body.

Laminae III and IV also contained pERK-positive cells that were not NK1r-immunoreactive, and in some cases these had dendrites that entered the superficial dorsal horn (Fig. 2). These appeared to be more common after the pinch stimulus than after either noxious heat or formaldehyde injection.

## Discussion

Although several studies have used immunocytochemistry to reveal the distribution of neurons in the spinal cord that contain pERK after various forms of noxious stimulation, few of these have attempted to identify the types of neuron that were pERK-positive. Here we show that virtually all of the large lamina III/IV NK1r-expressing neurons in the somatotopically appropriate part of the ipsilateral dorsal horn develop pERK-immunoreactivity within 5 mins of a noxious mechanical, thermal or chemical stimulus. Phosphorylation of ERK is thought to play an important role in the central sensitisation of dorsal horn neurons and in the development of inflammatory pain states [21-25]. Since the large lamina III/IV NK1r neurons provide a strong monosynaptic connection from substance P-containing (nociceptive) afferents to brain regions involved in pain perception, such as the lateral parabrachial area and thalamus [13,15,18], it is likely that phosphorylation of ERK in these cells plays a significant part in inflammatory pain.

Internalisation of the NK1 receptors on dorsal horn neurons has been demonstrated after several types of noxious stimulus applied to anaesthetised animals [6,32-34]. However, these studies concentrated on lamina I neurons or unidentified dendrites in lamina I. Internalisation of the receptor on the dorsal dendrites of lamina III NK1r-expressing neurons has been demonstrated after injection of capsaicin [6] or formalin [34] into the ipsilateral hindpaw. Our finding that the dorsal dendrites of these cells also have internalised receptors after noxious mechanical and thermal stimulation, together with the demonstration that ERK is phosphorylated after these types of stimulus, indicates that the large lamina III/IV NK1r-immunoreactive cells respond to a wide variety of noxious stimuli.

Previous studies have identified several neurotransmitters/neuromodulators and receptors that may be coupled to phosphorylation of ERK in the dorsal horn. These include glutamate, acting through NMDA [21,24,25,29], AMPA [25,29], and group I metabotropic [22,24,25,29] receptors, substance P and the NK1r [25,29], and brain-derived neurotrophic factor (BDNF) acting via the TrkB receptor [24,27]. Since we used ketamine (an NMDA receptor antagonist) for anaesthesia, it is possible that there was some suppression of ERK phosphorylation in our experiments. However, we found that many cells in the dorsal horn were pERK-positive, including virtually all of the large NK1r-immunoreactive lamina III/IV neurons.

Substance P-containing nociceptive afferents are glutamatergic [35] and make numerous asymmetrical synapses on these cells [18]. We have shown that all peptidergic afferents in the superficial dorsal horn are associated with synaptic AMPA receptors [36], and it is likely that NMDA receptors are also present at these synapses. Torsney and MacDermott [19] recorded from lamina III neurons that expressed NK1 receptors in spinal cord slices, but failed to detect Aδ/C fibre-mediated glutamatergic EPSPs on most of these cells. However, as the authors suggest [19], this may be because the recorded neurons were not those with long dorsal dendrites, or because their afferent input was not retained during the slice preparation. Group I metabotropic glutamate receptors include mGluR1 and mGluR5. Although both of these are present in the dorsal horn, it is unlikely that either is expressed by the lamina III/IV NK1r-immunoreactive neurons. mGluR5 is found at high density in laminae I-II, and at somewhat lower levels in lamina III, but appears to be associated only with small neurons [37,38]. mGluR1a is present on dendrites throughout laminae III-VI, but is virtually absent from laminae I and II [38], where the dendrites of these NK1r-expressing neurons have extensive arborisations. Although there have been reports of mGluR1a staining in the superficial dorsal horn [37] this is thought to represent a cross-reactivity of a commercially available mGluR1a antibody with mGluR5 [38]. Less is known about mGluR1b, but it is apparently expressed at relatively low levels in the spinal cord and does not seem to be present on large lamina III/IV neurons with prominent dorsal dendrites [38].

Both substance P and BDNF are also potential up-stream activators of pERK in these cells. The extensive receptor internalisation seen on the dorsal dendrites of most of the lamina III/IV NK1r-expressing neurons indicates that NK1rs on these cells were activated after each type of noxious stimulus. BDNF is contained in peptidergic afferent terminals in the superficial dorsal horn [39] and released following activation of C fibres [40]. In addition, it has been shown that the TrkB receptor is expressed by many neurons throughout the dorsal horn [27,41].

Further experiments with appropriate antagonists will be needed to determine which of these receptors is involved in phosphorylation of ERK in the lamina III/IV NK1r-expressing neurons.

## Conclusion

We have shown that virtually all of the large NK1r-immunoreactive neurons in laminae III/IV have phosphorylated ERK 5 minutes after three different types of noxious stimulation (mechanical, thermal or chemical). Since all of these cells are known to be projection neurons, our results suggest that cells of this type are involved in transmitting information from different types of noxious stimulus to the brain.

## Methods

### Animals and noxious stimulation

Ten adult male Wistar rats (Harlan, Loughborough, UK; 230 – 280 g) were used in this study. These were deeply anaesthetised with a mixture of ketamine and xylazine (7.33 and 0.73 mg/100 g i.p., respectively) and received one of the following three types of noxious stimulus: (1) immersion of the left hindpaw in water at 52°C for 60 secs (n = 3), (2) injection of 100 μl 2% formaldehyde into the left hindpaw (50 μl into the plantar pad and 10 μl into each digit; n = 3 rats), or (3) pinching of folds of skin at 12 points (6 each on dorsal and ventral surfaces of the left hindpaw, applied with forceps for 5 seconds at each point, n = 4). In each case the animals were maintained under general anaesthesia and perfused with 4% formaldehyde in 0.1M phosphate buffer under terminal pentobarbitone anesthesia 5 min after the end of the stimulus. All experiments were approved by the Ethical Review Process Applications Panel of the University of Glasgow, and were performed in accordance with the European Community directive 86/609/EC and the UK Animals (Scientific Procedures) Act 1986. All efforts were made to minimize the number of animals used and their suffering.

### Immunocytochemistry

The L4 spinal segment was removed from each animal and post-fixed in the same fixative at 4°C overnight. Parasagittal 60 μm sections from both sides of the segment were cut with a Vibratome, treated for 30 mins in 50% ethanol to enhance antibody penetration and processed for immunocytochemistry. The sections were incubated for 72 hours in a mixture of primary antibodies: mouse monoclonal antibody against phosphorylated ERK1/2 (Cell Signaling, Beverley, MA, USA, 1:1,000) and rabbit anti-NK1r (Sigma, Poole, Dorset, UK, 1:10,000) and then for 24 hours in species specific secondary antibodies conjugated to Alexa 488 (Invitrogen, Paisley, UK; 1:500) or Rhodamine Red (Jackson Immunoresearch, West Grove, PA, USA; 1:100). The pERK antibody detects both ERK1 and ERK2 that are dually phosphorylated at Thr202 and Tyr204 sites, and does not cross-react with JNK or p38 MAP kinase that are phosphorylated at the corresponding residues (Manufacturer's specification). For some sections an antibody raised against the C-terminal 14 amino acids of PKCγ was included, and this was detected with Cy5 anti-guinea pig IgG (Jackson Immunoresearch; 1:100). The PKCγ antibody has been shown to be specific as immunostaining is absent in the brain of PKCγ -/- mice [42].

All antibodies were diluted in PBS containing 0.3M NaCl and 0.3% Triton-X100 and incubations were carried out at 4°C. Sections were mounted in serial order in antifade medium (Vectashield, Vector Laboratories, Peterborough, UK) and stored at -20°C.

Sections were scanned with a Radiance 2100 or MRC 1024 confocal microscope (Bio-Rad, Hemel Hempstead, UK). All of the quantitative analysis was performed on sections scanned sequentially (to avoid fluorescent bleed-through) through a 20× lens.

### Analysis

Sections from the ipsilateral side were initially scanned through a 10× lens to reveal pERK-immunoreactivity and the 3 or 4 sections from each animal that showed maximal staining in the superficial laminae were selected for further analysis. These sections were invariably from the medial part of the dorsal horn which receives input from the regions that had been maximally stimulated. At this magnification it was possible to identify the band of pERK-immunoreactivity in the superficial dorsal horn, but individual pERK-positive neurons in deeper laminae were not well seen. The selection of sections was carried out before NK1r-immunostaining was viewed, in order to avoid bias towards sections that had pERK-immunoreactive NK1r-expressing neurons in laminae III-IV.

Selected sections were then examined through a 20× lens and all of the NK1r-immunoreactive neurons with cell bodies in laminae III or IV and dendrites that could be traced dorsally into lamina II (either in the same section or by following the dendrites through serial sections) were identified. Care was taken to avoid double-counting neurons with cell bodies that appeared on two adjacent sections. For all of the selected cells, the presence or absence of pERK staining in the soma and dendrites was recorded. In this way, we determined the proportion of lamina III/IV NK1r-immunoreactive cells in each animal that showed pERK-immunostaining.

## Competing interests

The authors declare that they have no competing interests.

## Authors' contributions

EP participated in the design of the study and the analysis; ADC and LMM carried out the immunocytochemistry and participated in the analysis; MW generated one of the antibodies and participated in writing the manuscript; AJT conceived of the study, participated in design and drafted the manuscript. All authors read and approved the final manuscript.
